# A Hybrid Vision Transformer-BiRNN Architecture for Direct k-Space to Image Reconstruction in Accelerated MRI

**DOI:** 10.3390/jimaging12010011

**Published:** 2025-12-26

**Authors:** Changheun Oh

**Affiliations:** Department of Software and Communication Engineering, Hongik University, Sejong 30016, Republic of Korea; choh@hongik.ac.kr

**Keywords:** accelerated MRI, bidirectional recurrent layers, MR image reconstruction, parallel MRI, RNN, vision transformer

## Abstract

Long scan times remain a fundamental challenge in Magnetic Resonance Imaging (MRI). Accelerated MRI, which undersamples k-space, requires robust reconstruction methods to solve the ill-posed inverse problem. Recent methods have shown promise by processing image-domain features to capture global spatial context. However, these approaches are often limited, as they fail to fully leverage the unique, sequential characteristics of the k-space data themselves, which are critical for disentangling aliasing artifacts. This study introduces a novel, hybrid, dual-domain deep learning architecture that combines a ViT-based autoencoder with Bidirectional Recurrent Neural Networks (BiRNNs). The proposed architecture is designed to synergistically process information from both domains: it uses the ViT to learn features from image patches and the BiRNNs to model sequential dependencies directly from k-space data. We conducted a comprehensive comparative analysis against a standard ViT with only an MLP head (Model 1), a ViT autoencoder operating solely in the image domain (Model 2), and a competitive UNet baseline. Evaluations were performed on retrospectively undersampled neuro-MRI data using R = 4 and R = 8 acceleration factors with both regular and random sampling patterns. The proposed architecture demonstrated superior performance and robustness, significantly outperforming all other models in challenging high-acceleration and random-sampling scenarios. The results confirm that integrating sequential k-space processing via BiRNNs is critical for superior artifact suppression, offering a robust solution for accelerated MRI.

## 1. Introduction

Magnetic Resonance Imaging (MRI) has established itself as an indispensable diagnostic tool in modern medicine, owing to its exceptional soft-tissue contrast and non-invasive characteristics. However, the inherent trade-off between image quality, spatial resolution, and acquisition time remains a fundamental challenge in clinical practice [1]. In particular, long scan times can increase patient discomfort and induce motion artifacts from involuntary movements such as breathing or heartbeat, which can severely compromise diagnostic accuracy. To address these problems, MRI acceleration involves intentionally undersampling k-space data and subsequently reconstructing a high-quality image from this incomplete information. The reconstruction process in accelerated MRI is intrinsically ill-posed, presenting infinite potential solutions due to the undersampling of k-space data, which has driven the development of diverse methodological approaches to address this inverse problem [2,3,4,5,6,7].

Early MRI acceleration techniques were based on explicit physical and mathematical models [6]. Parallel imaging (PI) methods such as SENSE (SENSitivity Encoding) [2] and GRAPPA (Generalized Autocalibrating Partially Parallel Acquisitions) [3] have been widely adopted in clinical settings, leveraging the spatial sensitivity information from multiple receiver coils to compensate for missing phase-encoding steps [4]. Whereas these methods have achieved significant acceleration factors, they are fundamentally limited by their linear reconstruction models and suffer from noise amplification (g-factor) at higher acceleration factors [8].

The advent of deep learning has revolutionized medical image reconstruction, offering data-driven approaches that can learn complex, non-linear mappings from undersampled data to fully sampled images [9]. In particular, Convolutional Neural Network (CNN) architectures, such as UNet, have shown remarkable success in MRI reconstruction by virtue of their excellent ability to capture local spatial features and hierarchical representations. However, due to the intrinsic nature of their local receptive fields, CNNs are inherently limited in modeling the long-range dependencies that are essential for removing aliasing artifacts across the entire image and understanding the global structural context [10,11]. As an alternative to overcome this limitation of CNNs, the Vision Transformer (ViT) has emerged [12,13,14]. The ViT treats an image as a sequence of patches and simultaneously models the global relationships among all patches using a self-attention mechanism. Thanks to this capability, the ViT demonstrates a strong advantage in understanding global context and has shown its potential in various medical image reconstruction problems. Recent research has demonstrated the potential of Transformer-based architectures in various medical imaging applications, including MRI reconstruction [10,11,15]. The ability of Transformers to process entire sequences in parallel, combined with their superior handling of long-range dependencies, makes them particularly well suited for the global nature of MRI reconstruction problems [11,16].

However, purely Transformer-based models often require massive datasets and lack the inductive bias to capture local high-frequency details effectively. To mitigate these limitations, hybrid architectures combining CNNs for local feature extraction and Transformers for global context modeling have recently attracted significant attention [17]. However, the majority of these hybrid approaches still operate predominantly in the image domain, treating k-space data merely as a constraint rather than exploiting their inherent sequential correlations for feature learning.

Therefore, to overcome this limitation, a hybrid architecture that operates in both the image and k-space domains is necessary. While the ViT addresses the image domain, an effective mechanism is required to model the k-space data, which fundamentally exhibit strong sequential correlations along their phase-encoding directions. On the other hand, Recurrent Neural Networks (RNNs) have been explored in MRI reconstruction to model the iterative nature of optimization algorithms or to capture dependencies in dynamic MRI sequences [18,19]. Unlike CNNs or ViTs, which treat data as static 2D or 3D volumes, RNNs are naturally suited for processing sequential data. Since MRI data acquisition in k-space is inherently a sequential process (i.e., line-by-line or shot-by-shot), RNN-based approaches offer a theoretical advantage in modeling the raw signal dependencies [7]. However, standalone RNN models often struggle with the high computational burden and may fail to capture complex spatial semantics once the data are transformed into the image domain.

The integration of Bidirectional Recurrent Neural Networks (BiRNNs) with Transformer architectures represents a novel approach to leveraging both sequential processing capabilities and global attention mechanisms. BiRNNs excel at capturing temporal dependencies in sequential data by processing information in both forward and backward directions, enabling the model to utilize future context when making predictions about current states. In the context of MRI reconstruction, k-space data exhibit inherent sequential characteristics that can benefit from bidirectional processing to extract domain-transformed latent representations.

This work aims to propose and evaluate an optimized ViT-based reconstruction model for parallel MRI that addresses the limitations of standard Transformer architectures for image reconstruction tasks. We introduce a novel autoencoder framework that combines the global attention capabilities of Vision Transformers with the sequential processing strengths of Bidirectional RNNs, specifically designed to handle the unique characteristics of k-space data in MRI reconstruction.

## 2. Methods

The ViT architecture was originally developed for classification tasks, typically concluding with an MLP that outputs a single label. However, this design is not well suited for image reconstruction, which requires precise spatial detail and dense pixel-wise prediction. Therefore, the final stage of the ViT must be restructured to accommodate reconstruction tasks.

In MRI, each image pixel is influenced by the entirety of k-space data—a property that initially motivated the use of fully connected layers in early reconstruction frameworks such as AUTOMAP. However, fully connected layers are computationally expensive due to the large number of parameters. To achieve a more efficient yet globally aware transformation, our method employs BiRNNs. A BiRNN consists of two RNNs processing the sequence in forward and reverse directions, enabling bidirectional context aggregation across the data. By applying BiRNN layers that alternately sweep horizontally and vertically over the 2D k-space, we construct a domain-transformed latent representation that preserves global structure while significantly reducing parameter complexity. In our approach, we introduce a ViT-based autoencoder architecture optimized for direct MRI reconstruction from undersampled k-space data. While the ViT encoder is leveraged to learn global contextual features from image patches, a key component of our design is the integration of BiRNNs for domain transformation from k-space to image space. To assess the effectiveness of our proposed design, we evaluate three architectures:

Model 1: The original ViT structure, in which the final MLP directly outputs the reconstructed image.
Model 2: A ViT-based autoencoder that includes a Transformer encoder–decoder pair but excludes any recurrent layers. The ViT encoder extracts patch-level features, and a Transformer decoder reconstructs the image [14].
Model 3 (Proposed): An enhanced autoencoder structure where the decoder is augmented with additional inputs consisting of folded images and domain-transformed latent features obtained from the BiRNN block. This integration of BiRNNs with the ViT enables the model to learn representations that reflect the sequential and global characteristics of k-space data.

**Figure 1:**
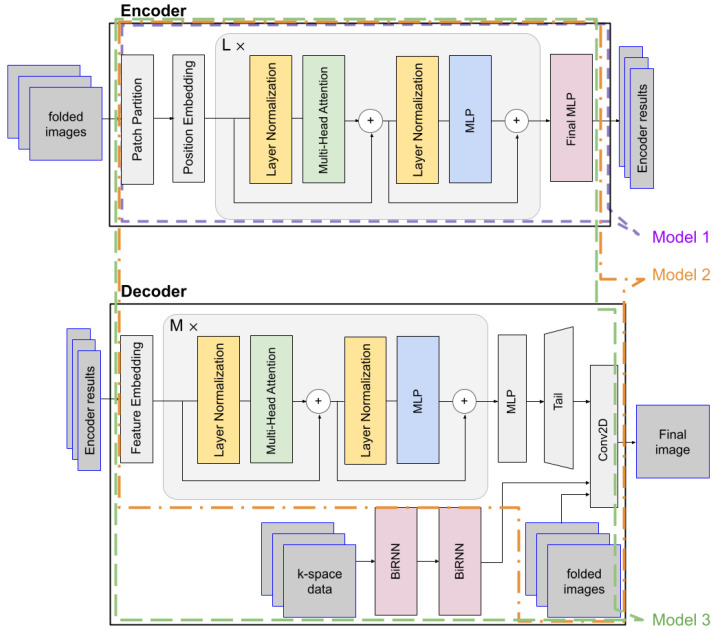
The architecture of the proposed ViT-based autoencoder.

Figure 1 illustrates the structure of the proposed Model 3, highlighting the nested configurations of Model 1 and Model 2. Model 1 corresponds to the encoder-only portion of Model 3 (indicated by the purple dotted line), while Model 2 shares the same encoder–decoder architecture as Model 3 but omits the BiRNN module.

For each model, the following experimental parameters were used: For Model 1, we applied the ‘ViT-Huge’ parameters [12]: layers = 32, hidden size = 1280, MLP size = 5120, heads = 16. Model 2 used the ‘ViT-Large’ parameters [12] for both the encoder and decoder: layers = 24, hidden size = 1024, MLP size = 4096, heads = 16. Model 3 used the ‘ViT-Base’ parameters [12] for the encoder. For the decoder and the BiRNNs, the following parameter settings were used: layers = 12, hidden size = 1280, MLP size = 5120, heads = 8, BiRNN hidden size = 384 × 10. According to the specified parameter settings, we can report the following model sizes: Model 1 contains 940,579,877 trainable parameters in total. Model 2 contains 643,630,553 parameters in total, including 337,035,240 in the encoder and 302,237,696 in the decoder. For Model 3, the network comprises 1,024,630,973 parameters in total, with 111,143,144 in the encoder, 188,899,840 in the decoder, and 637,102,080 across the BiRNN modules.

Our proposed model (Model 3) is designed based on an encoder–decoder framework. The encoder is tasked with learning a potent latent representation from the input images, while the decoder utilizes this representation, along with supplementary k-space data, to reconstruct the final, high-fidelity image. The encoder processes a batch of folded input images, denoted as $x\in R^{N\times H\times W\times 2C}$, where *N* is the number of images, *H* and *W* are the height and width, respectively, and *C* is the number of Receive (R*x*) channels. Each input image is partitioned into a sequence of flattened 2D patches. These patches are then mapped to a latent D-dimensional embedding space through a trainable linear projection. A learnable position embedding is added to these patch embeddings to retain positional information. The token embeddings are then passed through a series of L standard Transformer blocks. Each block consists of a Multi-Head Self-Attention (MSA) module and a feed-forward Multilayer Perceptron (MLP). Layer Normalization (LN) is applied before each module, and a residual connection is employed after each module. Finally, the sequence of encoded tokens from the last Transformer block is processed by a final MLP layer to produce the encoder results. This result encapsulates the high-level features extracted from the input images. The whole process of the encoder can be simply described as

(1) $$z_{enc}=\Phi_{Enc}\left(x\right)$$

where $\Phi_{Enc}\left(\cdot \right)$ represents the ViT encoder, including patch embedding, position embedding, and MSAs. The output $Z_{enc}\in R^{N\times D}$ consists of *N* patch tokens with embedding dimension *D*.

The decoder is a hybrid architecture designed to synthesize the final image by integrating the learned features from the encoder with auxiliary data streams. It receives three inputs: the encoder results, k-space data, and the original folded images. The encoder results first pass through a feature embedding layer before being processed by M successive Transformer blocks, and the resulting features are then processed by an MLP and a tail module to produce the Transformer path’s final output. The process of the ViT decoder and Up-tail can be described as

(2) $$\begin{array}{cc} z_{dec} & =\Phi_{Dec}\left(z_{enc}\right) \end{array}$$

(3) $$\begin{array}{cc} I_{ViT} & =\Phi_{Tail}\left(\mathrm{Reshape}\left(\mathrm{MLP}\left(z_{dec}\right)\right)\right) \end{array}$$

where $\Phi_{Dec}\left(\cdot \right)$ represents the ViT decoder layers, MLP(·) projects the features to match the required output dimensions, and $\Phi_{Tail}\left(\cdot \right)$ represents the upsampling sequence to generate the image domain features $I_{ViT}\in R^{H\times W\times C_{ViT}}$.

**Figure 2:**
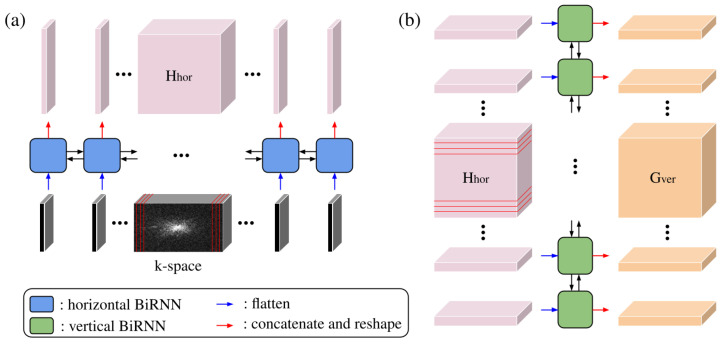
Schematic illustration of the bidirectional recurrent processing applied to k-space: (**a**) Horizontal BiRNN sweeps across each row to model left–right dependencies and generate the horizontal latent representation $H_{hor}$. (**b**) Vertical BiRNN processes $H_{hor}$ along the column direction to capture top–bottom correlations, producing the final latent representation $G_{ver}$.

Concurrently, the k-space data are processed by a sequence of two BiRNNs. This path is designed to capture contextual information from the frequency domain (k-space). Let $k\in R^{H\times W\times 2C}$ denote the input k-space; it is transformed into a sequence of column vectors $K=\{k_{1},k_{2},\ldots ,k_{W}\}$, where each $k_{i}$ represents the flattened vertical column at width index *i*. The process of the BiRNN can be described as

(4) $$\begin{array}{cc} {\vec{h}}_{i},{\overset{\leftarrow }{h}}_{i} & ={\mathrm{BiRNN}}_{hor}\left(k_{i},h_{i-1},h_{i+1}\right) \end{array}$$

(5) $$\begin{array}{cc} H_{hor} & =\left\{\left[{\vec{h}}_{1},{\overset{\leftarrow }{h}}_{1}\right],\left[{\vec{h}}_{2},{\overset{\leftarrow }{h}}_{2}\right],\ldots ,\left[{\vec{h}}_{W},{\overset{\leftarrow }{h}}_{W}\right]\right\}\in R^{W\times (2\times H\times D)} \end{array}$$

where ${\mathrm{BiRNN}}_{hor}\left(\cdot \right)$ represents the horizontally sweeping BiRNN layer (Figure 2a), which sequentially generates the k-space–image hybrid domain features ${\vec{h}}_{i},{\overset{\leftarrow }{h}}_{i}$. $H_{hor}$ represents the intermediate latent features, and *D* is the user-defined parameter.

To capture dependencies along the vertical axis (phase-encoding direction), the output of the horizontal sweep is rearranged as shown in Figure 2b. The intermediate feature tensor $H_{hor}$ is permuted and reshaped into a sequence of row vectors $V=\{v_{1},v_{2},\ldots ,v_{H}\}$, where each $v_{j}$ contains the flattened horizontal hidden features across the entire width for the *j*-th row.

(6) $$\begin{array}{cc} {\vec{g}}_{j},{\overset{\leftarrow }{g}}_{j} & ={\mathrm{BiRNN}}_{ver}\left(v_{j},g_{j-1},g_{j+1}\right) \end{array}$$

(7) $$\begin{array}{cc} G_{ver} & =\left\{\left[{\vec{g}}_{1},{\overset{\leftarrow }{g}}_{1}\right],\left[{\vec{g}}_{2},{\overset{\leftarrow }{g}}_{2}\right],\ldots ,\left[{\vec{g}}_{H},{\overset{\leftarrow }{g}}_{H}\right]\right\}\in R^{H\times (2\times W\times D)} \end{array}$$

(8) $$\begin{array}{cc} I_{RNN} & =\mathrm{Reshape}\left(G_{ver}\right)\in R^{H\times W\times 2D} \end{array}$$

where ${\mathrm{BiRNN}}_{ver}\left(\cdot \right)$ represents the vertically sweeping BiRNN layer, which sequentially generates the image domain features ${\vec{g}}_{j},{\overset{\leftarrow }{g}}_{j}$. $G_{ver}$ represents the output latent features, and *D* is the user-defined parameter. $I_{RNN}$ represents the image domain features, as a permuted and reshaped version of $G_{ver}$.

The outputs from the Transformer and BiRNN paths are fused with the original folded images to form a comprehensive, multi-modal representation. Finally, the refined features are combined and processed by a 2D convolutional layer, which maps this fused representation to the pixel space, generating the final reconstructed image as $\hat{y}=\mathrm{Conv}2D\left(\mathrm{Concat}\left(I_{ViT},I_{RNN},x\right)\right)$.

For the performance evaluation, we utilized neuro-MRI images from the ‘FastMRI’ dataset [20]. Specifically, T2w images acquired on a 3T MRI (Siemens, Skyra) with a matrix size of 384 (FE) × 396 (PE) × 16 (Rx) were used to train the model. Fully sampled k-space is available in FastMRI only for the training and validation sets. Thus, the models were trained on the training subset, and evaluation was carried out on the validation subset, which includes the fully sampled reference images needed for quantitative metrics. Undersampled data were retrospectively generated using two acceleration factors (AFs): R = 4 and R = 8. The R = 4 condition used 32 auto-calibration signal (ACS) lines, while the R = 8 condition used 16 ACS lines. For both acceleration factors, experiments were conducted using two different undersampling schemes: a regular Cartesian pattern and a random pattern that was newly generated for each sample. For random sampling, the remaining phase-encoding positions outside the ACS region were selected uniformly at random to achieve the desired acceleration factor. Quantitative assessment was conducted using three metrics: the normalized mean square error (nMSE), structural similarity index (SSIM) [21], and visual information fidelity (VIF) [22].

All models were trained using the Adam optimizer, with an initial learning rate of $1\times 10^{-4}$. A cosine annealing learning rate schedule was applied throughout training to gradually reduce the step size and stabilize convergence. No data augmentation strategies were used, as the focus of this study was to assess the intrinsic reconstruction capability of each architecture without introducing additional variability in the input distribution. All experiments were trained for 50 epochs on an NVIDIA TITAN RTX GPU, with each epoch requiring approximately 7 h to complete. The training loss was computed as the pixel-wise L1 loss between the reconstructed and reference images. For inference, the model processes a single slice in approximately 0.504 s, with a peak GPU memory consumption of 6.14 GB, demonstrating its computational feasibility for clinical workflows. We also reconstructed images using UNet (depth = 5, number of channels in the first convolutional layer = 64) and VarNet for comparison [23].

## 3. Results

**Figure 3:**
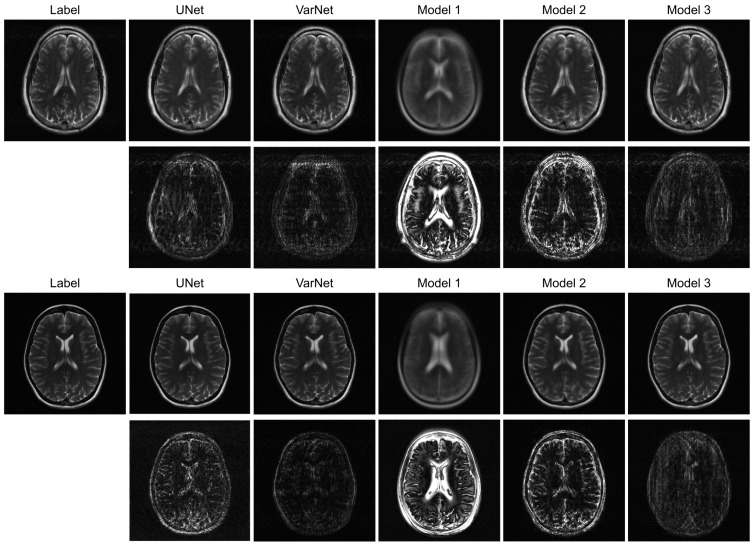
Reconstructed images and corresponding error maps for ViT-based models, UNet, and VarNet (intensity of the error maps is amplified by 10).

Figure 3 illustrates sample images and the corresponding error maps from Models 1–3, UNet, and VarNet for the R = 4 regular sampling pattern. The first column shows the reference (label) images, while columns 2–6 show the reconstructed images and the corresponding error maps (10× amplification) from UNet, VarNet, and Models 1, 2, and 3, respectively. Table 1 provides the quantitative results for the R = 4 regular sampling pattern.

A qualitative visual assessment of the reconstructed images in Figure 3 reveals significant performance differences among the models. The proposed Model 3 demonstrates exceptional reconstruction quality, achieving high fidelity to the reference image that is visually comparable to the performance of the UNet. Furthermore, the proposed model exhibits competitive performance against VarNet. As shown in Table 1, Model 3 achieves higher SSIM and VIF compared to VarNet, indicating superior perceptual quality and structural fidelity. The error map for Model 3 shows minimal structural error and low residual noise, indicating a successful recovery of fine anatomical details. In contrast, Model 1, which simply utilizes a final MLP for reconstruction, fails to capture essential anatomical structures, resulting in a severely blurred image with a high degree of structured error, as evidenced by its error map. Model 2, which employs a Transformer-based decoder, shows a substantial improvement over Model 1 by reconstructing the overall brain morphology. However, its error map contains more noticeable residual artifacts and noise compared to Model 3, suggesting a less complete removal of aliasing effects.

**Figure 4:**
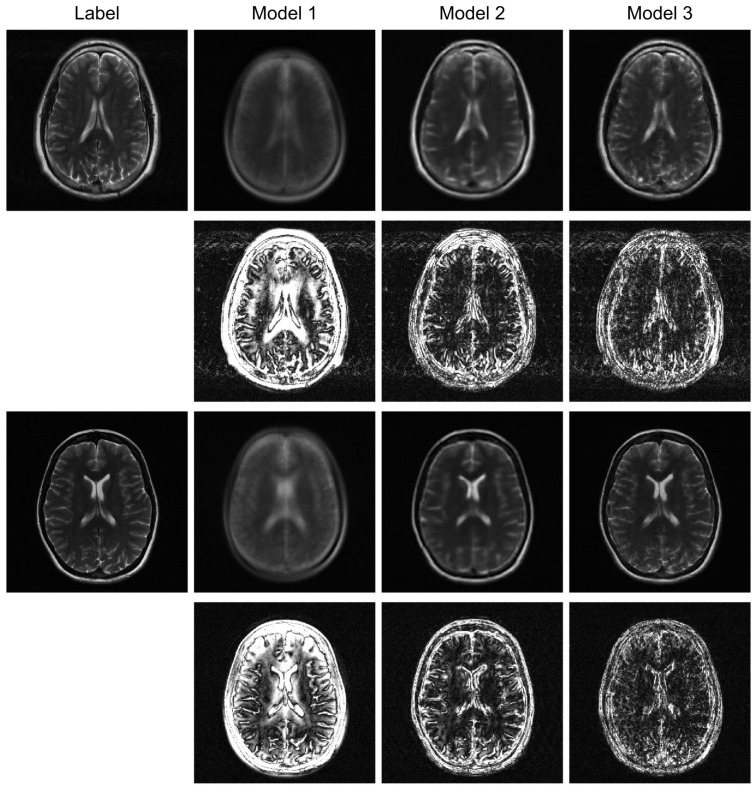
Qualitative evaluation on R = 4 randomly undersampled data. This figure compares the reconstruction outcomes and amplified (10×) error maps for Model 1, Model 2, and the proposed Model 3.

**Figure 5:**
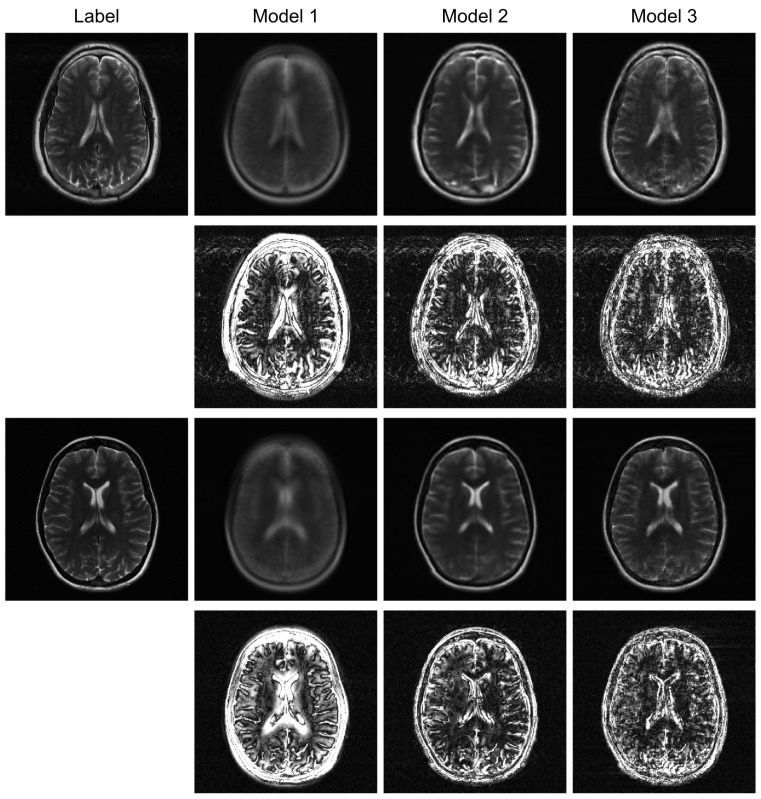
High-acceleration (R = 8) reconstruction with a regular sampling mask. The visual results and corresponding error maps (10× amplification) illustrate each model’s effectiveness in handling coherent aliasing.

**Figure 6:**
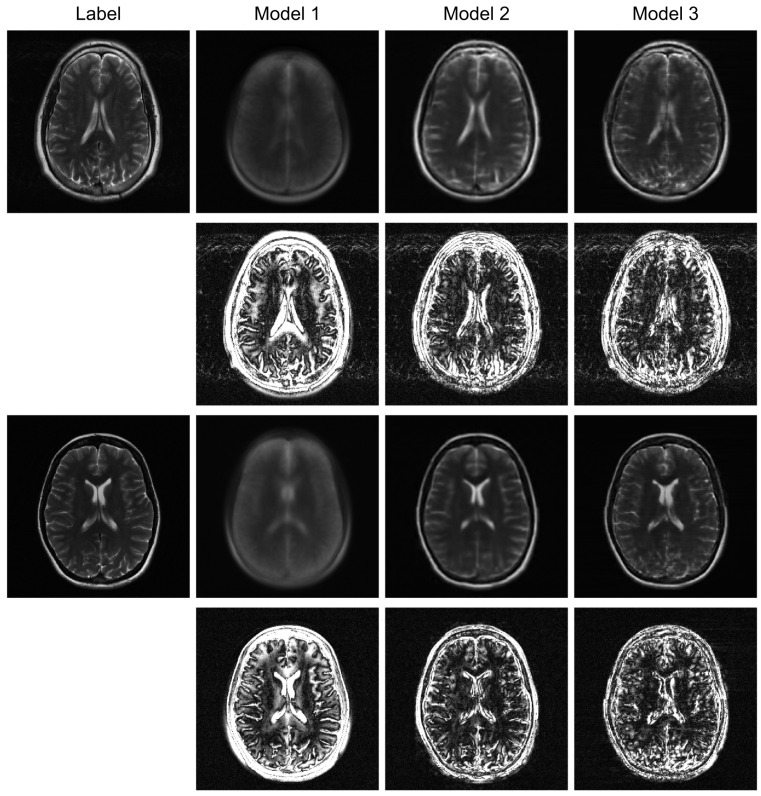
Robustness test under a high-acceleration (R = 8) random sampling condition. The reconstructed images and their error maps (10× amplification) demonstrate performance degradation and artifact patterns in a highly incoherent scenario.

To further evaluate the robustness of our proposed model, we conducted additional experiments under higher acceleration factors (R = 8) and with different undersampling patterns (regular and random). For the random sampling scenarios, a new undersampling mask was randomly generated for each sample, which prevents the model from overfitting to a fixed pattern and enhances generalization. The qualitative results are presented in Figure 4, Figure 5 and Figure 6, and the corresponding quantitative analysis is summarized in Table 2. At a higher acceleration factor of R = 8 (Figure 5 and Figure 6), the performance gap between the models becomes more pronounced. While all models exhibit increased artifacts compared to R = 4, Model 3 consistently preserves anatomical structures more effectively than the other models, particularly in the more challenging random sampling scenario (Figure 6). The quantitative results in Table 2 align with the visual assessment. Across all tested scenarios—R4 random, R8 regular, and R8 random—Model 3 consistently achieves the lowest nMSE and the highest SSIM and VIF scores.

## 4. Discussion

This study compares ViT-based image reconstruction models and introduces a ViT-based autoencoder with BiRNNs. The results show that a decoding Transformer improves reconstruction compared with an MLP decoder. To ensure a fair comparison, Models 1, 2, and 3 were set to a similar total parameter count. Model 3’s superior performance stems from its ability to leverage information from BiRNN structures effectively, achieving the highest efficiency by extracting domain-transformed latent representations from k-space. The hybrid, dual-domain architecture allows the model to synergistically process features from both the image and k-space domains. The ViT encoder captures global spatial context, which is crucial for structural coherence, while the BiRNNs model the sequential nature of k-space data, which is vital for removing complex aliasing artifacts. This dual-domain approach leads to higher reconstruction fidelity, especially in challenging high-acceleration scenarios.

The comparison between Model 2 and Model 3 effectively serves as an ablation study, isolating the contribution of the BiRNN module. The significant performance improvement observed in Model 3, particularly under high acceleration (R = 8) and random sampling conditions (Table 2), underscores the critical role of processing k-space data directly. While the Transformer decoder in Model 2 can reconstruct global structures from the encoder’s latent features, it struggles with the complex, non-local aliasing artifacts inherent in undersampled data. The BiRNN module in Model 3 addresses this by interpreting k-space as sequential data, effectively capturing the structured correlations along the phase-encoding directions. This allows the model to disentangle aliasing patterns from true anatomical features before the final fusion step, resulting in superior artifact suppression and detail preservation, as visually confirmed in Figure 4 and Figure 5.

Furthermore, the model’s robustness against different sampling patterns demonstrates its generalization capabilities. The regular sampling pattern (Figure 5) produces coherent, line-like artifacts, whereas the random pattern (Figure 4 and Figure 6) generates more incoherent, noise-like artifacts. Model 3’s consistent high performance in both scenarios indicates that the hybrid architecture, which processes global image-domain context via the ViT and models frequency-domain sequential dependencies via BiRNNs, achieves high adaptability to varying artifact structures. This adaptability is a significant advantage over methods optimized for only a specific type of artifact texture.

## 5. Conclusions

In this study, we have introduced a novel, hybrid, dual-domain deep learning architecture for accelerated MRI reconstruction. By combining a Vision Transformer-based autoencoder with Bidirectional Recurrent Neural Networks, our model effectively leverages both global image-domain features and sequential k-space information. Through comprehensive experiments across various acceleration factors and sampling patterns, we demonstrated that our proposed model significantly outperforms other ViT-based architectures and achieves competitive results. The results highlight the importance of integrating domain-specific knowledge, such as the sequential structure of k-space, into powerful deep learning frameworks. This work demonstrates the potential of hybrid Transformer–RNN models to advance the field of medical image reconstruction, paving the way for faster and more accurate MRI examinations.

## Figures and Tables

**Table 1:** Quantitative analysis of Models 1–3, UNet, and VarNet for the R = 4 regular sampling pattern. Performance is evaluated using nMSE, SSIM, and VIF (mean ± standard deviation).

	UNet	VarNet	Model 1	Model 2	Model 3
nMSE (%)	1.92±0.230	1.37±0.13	24.83±8.76	7.52±1.47	1.48±0.26
SSIM	0.888±0.010	0.888±0.036	0.691±0.060	0.791±0.025	0.890±0.012
VIF	0.954±0.073	0.912±0.095	0.134±0.116	0.755±0.051	0.982±0.037

**Table 2:** Quantitative analysis of Models 1–3 for R4 random, R8 regular, and R8 random. Performance is evaluated using nMSE, SSIM, and VIF (mean ± standard deviation).

Sampling	Metric	Model 1	Model 2	Model 3
R4 random	nMSE (%)	31.98±8.32	9.41±1.67	3.82±1.32
	SSIM	0.719±0.057	0.826±0.029	0.893±0.018
	VIF	0.048±0.037	0.500±0.091	0.748±0.041
R8 regular	nMSE (%)	29.13±7.28	11.21±2.23	9.33±2.22
	SSIM	0.727±0.049	0.820±0.031	0.830±0.031
	VIF	0.048±0.085	0.484±0.107	0.609±0.072
R8 random	nMSE (%)	34.40±6.06	15.42±3.11	9.74±3.82
	SSIM	0.718±0.052	0.802±0.037	0.831±0.039
	VIF	0.024±0.051	0.427±0.070	0.587±0.036

## Data Availability

The datasets presented in this article are not readily available due to time limitations. Requests to access the datasets should be directed to the author.
